# ST6Gal-I modulates docetaxel sensitivity in human hepatocarcinoma cells via the p38 MAPK/caspase pathway

**DOI:** 10.18632/oncotarget.10192

**Published:** 2016-06-21

**Authors:** Xixi Chen, Liping Wang, Yujie Zhao, Shiqi Yuan, Qiang Wu, Xiaoling Zhu, Bachir Niang, Shujing Wang, Jianing Zhang

**Affiliations:** ^1^ School of Life Science and Medicine, Dalian University of Technology, Panjin 124221, Liaoning, China; ^2^ Department of Biochemistry and Molecular Biology, Institute of Glycobiology, Dalian Medical University, Dalian 116044, Liaoning, China

**Keywords:** HCC, ST6Gal-I, docetaxel, apoptosis, p38 MAPK

## Abstract

The β-galactoside α2-6-sialyltransferase 1 (ST6Gal-I) is the principal sialyltransferase responsible for the addition of α2-6-sialic acid to the termini N-glycans on cell surface. Although ST6Gal-I in cancer cell resistance to chemotherapeutics agents has been previously reported, the role of ST6Gal-I in clinical drug resistance of hepatocellular carcinoma (HCC) is not fully understood. In this study, we found that knockdown of ST6Gal-I increased the sensitivity of hepatocarcinoma MHCC97-H cells to docetaxel treatment by instigating the process of apoptosis. Silencing ST6Gal-I expression decreased the survival rate of MHCC97-H cells after docetaxel treatment. Importantly, ST6Gal-I silencing resulted in an increasing of phospho-p38, Bax, Bad, cytochrome c and the cleaved caspase-9, 3 and PARP, while a decreasing of the anti-apoptotic protein Bcl-2. In addition, we found that p38 MAPK and caspase-3 inhibitors can reduce the enhanced apoptosis levels of MHCC97-H cells resulted by either ST6Gal-I silencing or docetaxel treatment. Conversely, exogenous expression of ST6Gal-I in hepatocarcinoma Huh7 cells inhibited apoptotic cell death and prevented docetaxel-induced apoptosis by inhibiting p38 MAPK mediated mitochondrial-dependent pathway. Taken together, these results indicate that ST6Gal-I might play a positive role in mediating the survival of human hepatocarcinoma cells and could be a potential target for gene and antitumor drugs therapy.

## INTRODUCTION

Liver cancer (mainly hepatocellular carcinoma, HCC) is the second most frequent cause of death from cancer (745,000 deaths in 2012) in the world [1]. It is a leading cancer in developing countries where 649,000 new cases were estimated in 2012, especially in China (50% of the total) [2]. Despite recent advances in chemotherapy and surgical resection during the treatment of HCC, its prognosis still remains poor due to tumors recurrence and metastasis [3]. The frequency of chemotherapeutic drugs resistance has inspired researchers to focus on new molecular mechanisms which can be potentially involved in the pathogenesis and treatment of HCC [4].

The β-galactoside α2-6-sialyltransferase 1 ST6Gal-I can catalyze the transfer of sialic acid from CMP-sialic acid to the termini Galβ1-4GlcNAc units of N-glycans [5]. ST6Gal-I is known to be highly expressed in multiple types of cancer, including colorectal, ovarian, oral, breast, epithelial and liver carcinomas [6–11], and its expression has been positively correlated with adhesion, invasion and metastasis in tumor cells [12–14]. Recently it has been reported that ST6Gal-I mediates the sialylation of tumor-necrosis factor receptor-1 (TNFR1) and Fas receptors to inhibit cell apoptosis, and plays a vital role in resistance to cisplatin-induced apoptosis of ovarian cancer [15, 16, 7]. However, the relationship between ST6Gal-I and clinical drug resistance of HCC still remains poorly understood.

Docetaxel (DTX) is a semisynthetic analogue of taxane, extracted from needles of European yew. It was reported to be involved in the mechanisms inhibiting microtubules disassembly as well as the enhancement of tubulin polymerization [17, 18]. Docetaxel had shown effective actions either alone or in combination with other chemotherapeutic drugs during the treatment of various types of cancer including prostate, ovarian, breast, non-small cell lung and liver cancers [19–23]. Taking the fact that ST6Gal-I is significantly overexpressed in liver cancer, and its important role in tumor cell apoptosis, we aimed to determine whether its expression could impact the sensitivity of hepatocarcinoma cells to docetaxel. Nevertheless, due to the lack of specificity into tissues and cells, the challenge lies into the pretreatment of docetaxel-resistant advanced HCC [24].

In this study, we analyzed the role of ST6Gal-I in the apoptosis and sensitivity of hepatocarcinoma cells to docetaxel, and explored the possible molecular mechanisms involved in the regulation of tumor cells apoptosis and chemosensitivity. The results showed that ST6Gal-I knockdown increased the sensitivity of hepatocarcinoma MHCC97-H cells to docetaxel, and ST6Gal-I overexpression inhibited the docetaxel-induced apoptosis in Huh7 cells. In addtion, ST6Gal-I silencing resulted in the activation of p38 MAPK signaling pathway, while a decreasing of the anti-apoptotic protein Bcl-2. Conversely, ST6Gal-I overexpression in Huh7 cells inhibited p38 MAPK mediated mitochondrial-dependent pathway. Together, this study indicates that ST6Gal-I could modulates docetaxel sensitivity in human hepatocarcinoma cells via the p38 MAPK/caspase pathway, and might be a potential target for gene and antitumor drugs therapy.

## RESULTS

### Silencing ST6Gal-I expression induces apoptosis and enhances sensitivity of MHCC97-H cells to docetaxel

**Figure 1:**
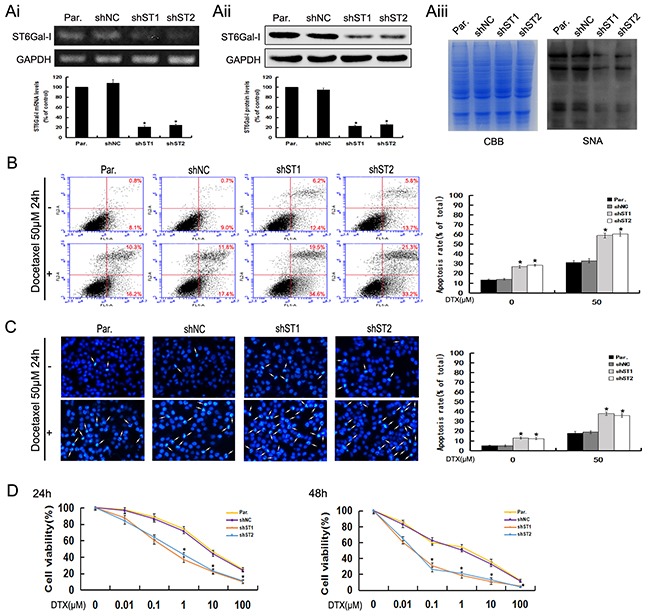
ST6Gal-I knockdown induces apoptosis and increases the sensitivity of MHCC97-H cells to docetaxel **Ai-Aiii.** ST6Gal-I was stably knocked down in MHCC97-H cells, the mRNA (Ai) and protein (Aii) levels of ST6Gal-I were determined by RT-PCR and Western-blot assays and normalized for GAPDH (*P<0.05). The α2,6-linked sialic acid (Aiii) levels were determined by SNA lectin staining. Coomassie Brilliant Blue (CBB) staining was used to normalize the protein amounts. **B.** The rates of apoptosis were determined by flow cytometry analysis of Annexin V-FITC/PI. Results are representative of three independent experiments (*P<0.05). **C**. Representative images of DAPI staining. Results are representative of 10 different fields (×100) from three independent experiments (*P<0.05). **D.** Cell viability following docetaxel treatment was detected by CCK8 assay. Note that the (1, 10, 100μM for 24h and 0.1, 1, 10, 100μM for 48h) measurements were significantly different (*P<0.05). Results represent the mean +/− SD of triplicate wells and are representative of at least three independent experiments. Par., MHCC97-H; shNC, Negative Control-shRNA transfectants; shST1 and shST2, ST6Gal-I-shRNA transfected stable clones.

To explore the potential role of ST6Gal-I in hepatocellular carcinoma cells, we used shRNA to silence the expression of ST6Gal-I in MHCC97-H cells which have higher expression level of ST6Gal-I. The knockdown efficiency was analyzed by RT-PCR, Western-blot and Lectin-blot assays. The results demonstrated that ST6Gal-I-shRNA transfection in MHCC97-H significantly decreased the expression of ST6Gal-I in mRNA, protein and glycans levels (*P<0.05) (Figure 1Ai, Aii and Aiii).

Two stable knockdown cell lines and control cells were treated with docetaxel to study the possible functions of ST6Gal-I in modulating the apoptosis and chemosensitivity in hepacellular carcinoma cells. Using Annexin V-FITC/PI staining, we found that the apoptosis rates of ST6Gal-I knockdown cells increased statistically as compared to the control cells in the presence or absence of docetaxel (Figure 1B). Furthermore, we observed more marked morphological changes in ST6Gal-I knockdown cells (Figure 1C). These results indicate that silencing ST6Gal-I can increase the sensitivity of MHCC97-H cells to docetaxel treatment.

To determine whether the enhanced apoptosis in ST6Gal-I knockdown cells combined with docetaxel treatment is reflected at the level of cell survival, we performed a colorimetric cell viability (CCK8) assay. We found that the ST6Gal-I knockdown cells were less resistant to docetaxel than control cells when exposed to different concentrations for 24h and 48h (Figure 1D). These results suggest that ST6Gal-I knockdown leads to reduced cell survival of MHCC97-H cells following docetaxel treatment.

### ST6Gal-I silencing sensitizes MHCC97-H cells to docetaxel-induced apoptosis through potentiation of p38 MAPK mediated mitochondrial-dependent pathway

**Figure 2:**
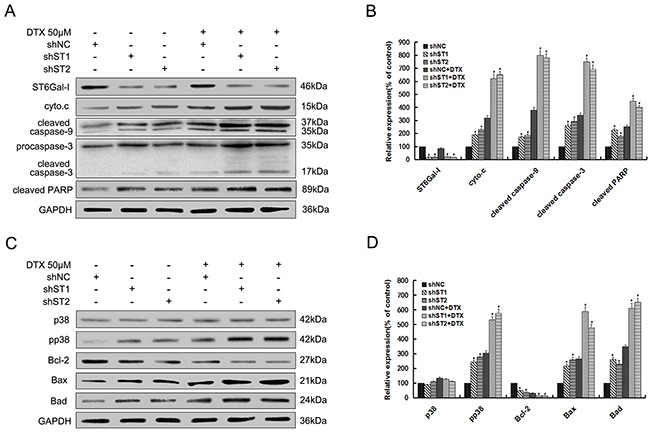
Silencing of ST6Gal-I in MHCC97-H cells enhances docetaxel-induced apoptosis through potentiation of p38 MAPK mediated mitochondrial-dependent pathway Par. shNC and shST1/2 cells were treated with the presence or absence of 50μM docetaxel for 24h. **A.** ST6Gal-I, cytochrome c, cleaved forms of caspase-9, 3 and PARP protein levels were determined by Western-blot. **C.** Bcl-2 family, p38 and pp38 protein levels were determined by Western-blot. **B** and **D.** Quantification of protein levels was performed by densitometry. Results represent the mean +/− SD of the expression levels from three independent experiments standardized to GAPDH expression and normalized to 100% in Par. cells without docetaxel treatment. (*P<0.05 compared to Par. cells without docetaxel, ^#^*P<0.05* compared to Par. cells with docetaxel).

To investigate the molecular events involved in docetaxel-induced apoptosis, we checked the role of ST6Gal-I knockdown in the activation of the caspase cascade. The expression of cytochrome c and the cleaved caspase-9, 3 and PARP were investigated by Western-blot analysis. As shown in Figure 2A and 2B, their expression levels significantly increase in ST6Gal-I knockdown cells either alone or in combination with docetaxel treatment. Meanwhile, no apparent changes in the expression of ST6Gal-I were observed after docetaxel treatment.

The role of caspases in the apoptotic cascade have been linked to the activation of the p38 MAPK pathway [25], we therefore examined the role of ST6Gal-I silencing in this pathway. As shown in Figure 2C and 2D, there was no significant difference in the p38 expression between different groups. However, phosphorylated p38 (pp38) expression was significantly increased in ST6Gal-I knockdown cells, for both untreated and treated with docetaxel. We also checked the expression of Bcl-2 family proteins, and found that ST6Gal-I silencing induces a decrease in the expression of Bcl-2 while the expression of Bax and Bad increased significantly.

Together, these findings suggest that ST6Gal-I silencing could enhanced docetaxel-induced apoptosis, which might be attributed to the potentiation of p38 MAPK mediated mitochondrial-dependent pathway.

### Caspase-3 and p38 inhibition reduces the enhanced apoptosis of MHCC97-H cells resulted by ST6Gal-I silencing or docetaxel treatment

**Figure 3:**
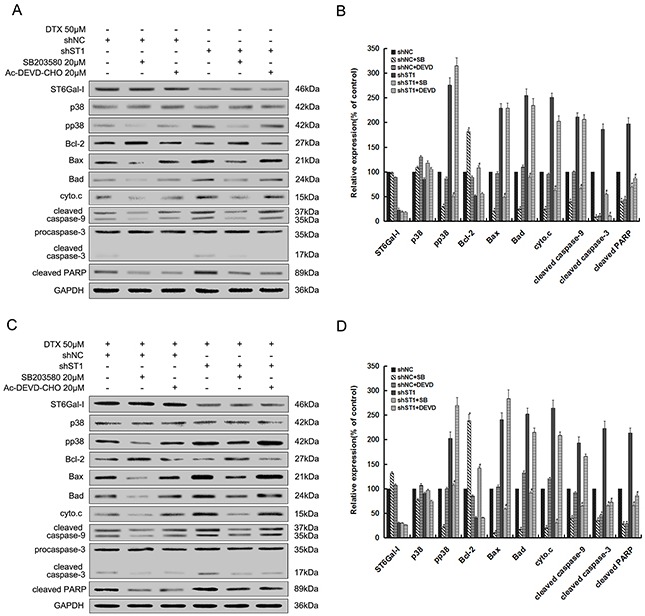
Inhibition of p38 MAPK pathway and caspase-3 activity decreases ST6Gal-I silencing or docetaxel-induced MHCC97-H cells apoptosis shNC and shST1 cells were stimulated with 0μM or 50μM of docetaxel for 24 h after pretreatment with SB203580 (20μM) or Ac-DEVD-CHO (20μM) for 30 minutes. **A** and **C.** Protein levels were determined by Western-blot. **B** and **D.** Quantification of protein levels was performed by densitometry. Results represent the mean +/− SD of the expression levels from three independent experiments standardized to GAPDH expression and normalized to 100% in shNC cells without inhibitor. (*P<0.05 compared to shNC cells without inhibitor, ^#^*P<0.05* compared to shST1 cells without inhibitor).

To further confirm the results aboved, we pretreated ST6Gal-I knockdown cells (shST1) and Negative Control-shRNA cells (shNC) with the specific p38 MAPK pathway inhibitor (SB203580) and selective inhibitor of caspase-3 (Ac-DEVD-CHO) before exposure to 0μM or 50μM docetaxel. We found that in the presence or absence of docetaxel, after treatment with SB203580, the protein levels of pp38, proapoptotic members Bax and Bad, cytochrome c, the cleaved caspase-9, 3 and PARP were inhibited, while the expression of Bcl-2 increased significantly. However, addition of Ac-DEVD-CHO only decreased the protein levels of cleaved caspase-3 and PARP, and there are no apparent changes observed in the expression of ST6Gal-I and p38 (Figure 3). Therefore, these findings provide more convincing information about the downstream pathway underlining the ST6Gal-I regulatory network.

### ST6Gal-I overexpression protects hepatocarcinoma Huh7 cells from docetaxel-induced apoptosis

**Figure 4:**
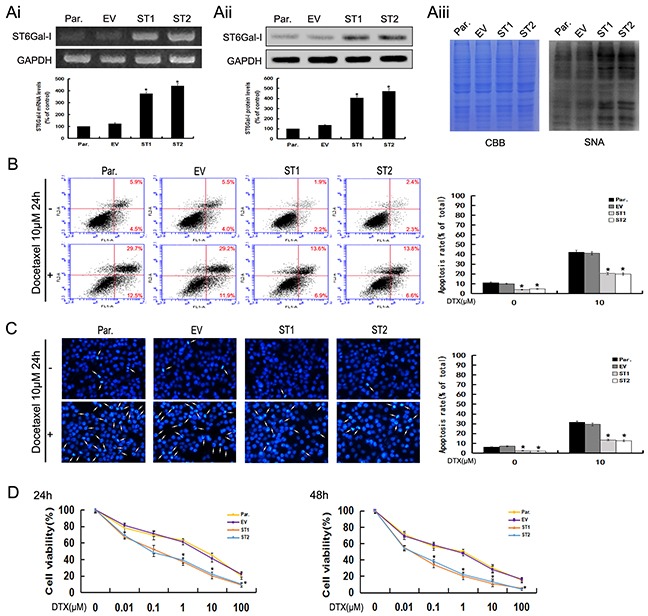
Upregulation of ST6Gal-I increases the survival rate of Huh7 cells and protects Huh7 cells from docetaxel-induced apoptosis **Ai-Aiii.** ST6Gal-I was stably over-expressed in Huh7 cells, the mRNA (Ai) and protein (Aii) levels of ST6Gal-I were determined by RT-PCR and Western-blot assays and normalized for GAPDH (*P<0.05). The α2,6-linked sialic acid (Aiii) levels were determined by SNA lectin staining. Coomassie Brilliant Blue (CBB) staining was used to normalize the protein amounts. **B.** The rates of apoptosis were determined by flow cytometry analysis of Annexin V-FITC/PI. Results are representative of three independent experiments (*P<0.05). **C.** Representative images of DAPI staining. Results are representative of 10 different fields (×100) from three independent experiments (*P<0.05). **D.** Cell viability following docetaxel treatment was detected by CCK8 assay. Note that the (1, 10, 100μM for 24h and 0.1, 1, 10, 100μM for 48h) measurements were significantly different (*P<0.05). Results represent the mean +/− SD of triplicate wells and are representative of at least three independent experiments. Par., Huh7; EV, Empty vector transfectants; ST1 and ST2, pcDNA3.1/ST6Gal-I vector transfected stable clones.

To further confirm the role of ST6Gal-I in the apoptosis of hepatocarcinoma cells, we used pcDNA3.1/ST6Gal-I overexpression vector to upregulate the level of ST6Gal-I expression in Huh7 cells. A significant increase in ST6Gal-I expression at mRNA, protein and glycans levels after pcDNA3.1/ST6Gal-I transfection were observed by RT-PCR, Western-blot and Lectin-blot assays (*P<0.05) (Figure 4Ai, Aii and Aiii). By using Annexin V-FITC/PI and DAPI staining, we found that the apoptosis rates of ST6Gal-I overexpressed cells decreased statistically compared to control cells in the presence or absence of docetaxel (Figure 4B and 4C).In addition, we also found that ST6Gal-I overexpressed cells were more resistant to docetaxel than control cells when exposed to different concentrations for 24h and 48h (Figure 4D). Together, these results indicate that upregulation of ST6Gal-I could protect Huh7 cells from docetaxel-induced apoptosis and increase the survival rate of Huh7 cells following docetaxel treatment.

### ST6Gal-I overexpression inhibits the p38 MAPK/caspase signaling pathway in Huh7 cells

**Figure 5:**
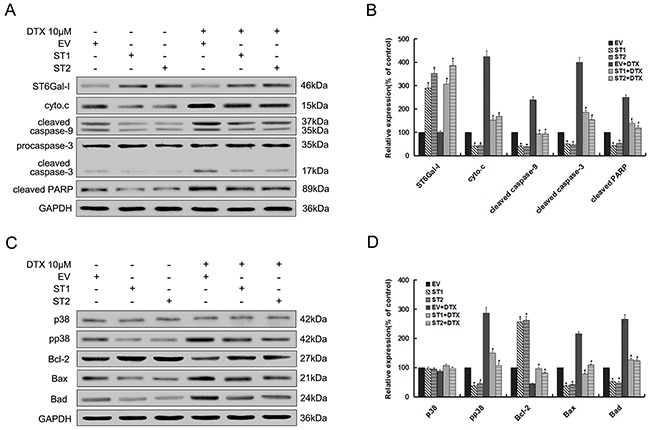
Upregulation of ST6Gal-I protects Huh7 cells from docetaxel-induced apoptosis through inhibition of p38 MAPK mediated mitochondrial-dependent pathway Par. EV and ST1/2 cells were treated with the presence or absence of 10μM docetaxel for 24h. **A.** ST6Gal-I, cytochrome c, cleaved forms of caspase-9, 3 and PARP protein levels were determined by western blot. **C.** Bcl-2 family, p38 and pp38 protein levels were determined by western blot. **B** and **D.** Quantification of protein levels was performed by densitometry. Results represent the mean +/− SD of the expression levels from three independent experiments standardized to GAPDH expression and normalized to 100% in Par. cells without docetaxel treatment. (*P<0.05 compared to Par. cells without docetaxel, ^#^*P<0.05* compared to Par. cells with docetaxel).

To examine whether ST6Gal-I overexpression could affect the p38 MAPK mediated mitochondrial-dependent pathway, we analyzed the expression levels of related proteins in this pathway by Western-blot assays. The results showed that cytochrome c and the cleaved caspase-9, 3 and PARP levels significantly decreased in ST6Gal-I overexpressed cells compared to control cells either alone or in combination with docetaxel treatment (Figures 5A and 5B). Meanwhile, there was no significant difference in the ST6Gal-I expression after docetacel treatment. As shown in Figure 5C and 5D, pp38, Bax and Bad expression levels decreased, and Bcl-2 increased in ST6Gal-I overexpressed cells relative to control cells before and after treatment with docetaxel. However, there was no significant difference in the p38 expression between the groups. Thus, ST6Gal-I overexpression could protects Huh7 cells from docetaxel-induced apoptosis, which might be attributed to the inhibition of p38 MAPK mediated mitochondrial-dependent pathway.

## DISCUSSION

Aberrant sialylation have been reported to correlate with the invasion and metastasis of tumor cells [13, 14]. A study from Gu's group showed that ST6Gal-I contributes to transforming growth factor-β-dependent epithelial-mesenchymal transition (EMT) [26]. Nowadays cancer associated-glycans have become the targets of anticancer drugs [27], however the mechanisms through which ST6Gal-I silencing could be benefit during diagnostic or chemotherapies are not fully investigated. In this study, we found that ST6Gal-I knockdown increased the sensitivity of hepatocarcinoma MHCC97-H cells to docetaxel treatment by instigating the process of apoptosis and decreasing the survival rate. By contrast, exogenous expression of ST6Gal-I could protect hepatocarcinoma Huh7 cells from docetaxel-induced apoptosis. These observations clearly indicate that the changes in ST6Gal-I expression levels may consequently affect the biological functions of tumor cells such as apoptosis and survival.

Many cellular stress signaling pathways have been involved in the apoptosis machinery and most of them are executed in an orderly manner. Among the different molecular regulations, the caspase cascade represents a key system. Our results have emphasized a regulatory network where ST6Gal-I facilitates the docetaxel-induced apoptosis through a mitochondrial pathway with the release of cytochrome c which in turn activates caspase-9 [28]. Consequently, caspase-9 affects the activation of downstream caspase-3 which cleaves the target proteins like PARP leading to DNA damage and cell death [29]. Bcl-2 family members are known to play an integral role in the regulation of caspases activation and in the mitochondrial apoptosis pathway [30]. This might explain the reason why the increasing levels of the pro-apoptotic proteins (Bad and Bax) and the decreasing level of anti-apoptotic protein (Bcl-2) were observed in the ST6Gal-I knockdown cells. Therefore, ST6Gal-I may be a potential and valuable target for the clinical treatment of hepatocellular carcinoma.

Docetaxel plays a vital role in the regulation of Bcl-2 phosphorylation, which might through the action of kinases in tumor cells [31]. Among the various kinases pathway, the p38 mitogen-activated protein kinases (p38 MAPK) plays a key role in signal transduction and other cellular processes. Previous studies have reported that p38 MAPK pathway participate in docetaxel-induced apoptosis in prostate cancer cells, and it may play both the role of activator and effector for the caspase cascade [32, 33]. Here, we observed that knockdown/overexpression of ST6Gal-I increase/decrease the p38 phosphorylation, highlighting its instigating role in the modulation of the apoptotic network through the p38 MAPK pathway. In addition, theses finding were confirmed by using SB203580 and Ac-DEVD-CHO inhibitors of the p38 MAPK pathway and caspase-3 respectively which induce a decrease in the expression of the proteins localized in the downstream of the different pathways.

Although not fully understood, the roles of α2,6 sialylation in cancer progression may be exerted by affecting the structures of specific sialylated glycoproteins [34]. It has been reported that β1 intergrin, CD45, epidermal growth factor receptor (EGFR), TNFR1 and Fas death receptors are the target proteins for ST6Gal-I [35–37, 15, 16]. ST6Gal-I expression is well known to be modulated at the transcript level and positively regulated by oncogenic ras [38, 39]. In spite of the fact that many proteins involved in the regulation of ST6Gal-I transcription, the specific substrates of ST6Gal-I and the information regarding how ST6Gal-I is regulated by ras during docetaxel induced-apoptosis in hepatocarcinoma cells still deserve further study.

In summary, ST6Gal-I is involved in the apoptosis and survival of hepatocarcinoma cells by the modulation of p38 MAPK mediated mitochondrial-dependent pathway. Therefore, a better understanding of ST6Gal-I regulation and role in the treatment and diagnosis of hepatocarcinoma may lead to new antitumor strategies that will sensitize unresponsive liver cancers to docetaxel-based chemotherapy.

## MATERIALS AND METHODS

### Cell culture

Human hepatoma carcinoma cell line MHCC97-H was purchased from Liver Cancer Institute (Zhongshan Hospital, Fudan University, Shanghai, China). Established HCC cell line Huh7 was purchased from the Cell Bank of CAS (Shanghai, China). Cells were maintained in Dulborcco's modified Eagle's medium (DMEM)(Gibco) and supplemented with 10% fetal bovine serum (FBS) (Gibco). All cells were cultured under 5% CO2 at 37°C.

### Construction of vectors and establishment of stable transfectants

The CDS region of ST6Gal-I (1218 bp) was cloned into the pcDNA3.1 (−) vector to construct pcDNA3.1/ST6Gal-I. The sequences of the primer pairs were as follows: Forward, 5′-GGGCCCATGATTCACACCAACCTGAAG-3′ (*Apa* I); Reverse, 5′-CTCGAGG CAGTGAATGGTCCGGAAGCC-3′ (*Xho* I). Short hairpin RNA (shRNA) specific for ST6Gal-I (ST6Gal-I-shRNA) and Negative Control-shRNA (NC-shRNA) were obtained from GenePharma (Shanghai, China). The sequences of the short hairpin RNA were as follows, ST6Gal-I-shRNA: 5′-CACCGTACCAGAATCCGGATTATTTCAAGAGAATAATCCG GATTCTGGTACTTTTTTG-3′ and 5′-GATCCAAAAAAGTACCAGAATCCGGATTATTCTCTTGA AATAATCCGGATTCTGGTAC-3; NC-shRNA:5′-CAC CGTTCTCCGAACGTGTCACGTCAAGAGATTACGT GACACGTTCGGAGAATTTTTTG-3′ and 5′-GAT CCAAAAAATTCTCCGAACGTGTCACGTAATCTCTT GACGTGACACGTTCGGAGAAC. Those shRNAs were annealed and ligated into pGPU6 vector to generate shRNA constructs (ST6Gal-I-shRNA, NC-shRNA). Cells were transfected with the mixture of ST6Gal-I-shRNA or NC-shRNA or pcDNA3.1/ST6Gal-I or pcDNA3.1 and Lipofectamine2000^TM^ (Invitrogen) according to the manufacturer's instraction. After 48h, the culture medium was replaced with complete medium containing 800 mg/ml of G418 (Sigma-Aldrich) for selecting stably transtected cells. Stable clones were isolated and expanded for further studies.

### RT-PCR

Total RNA was extracted using the RNAiso Reagent Plus (Takara), and complementary DNA (cDNA) was synthesized using RT-PCR kit (Takara) according to the manufacturer recommendations. The sequences of the primer pairs were as follows: for ST6Gal-I, 5′-AGCCCTTTTACATCCTCAAG-3′ (forward) and 5′-ATGATGATACCAAGCATCCC-3′ (reverse); for glyceraldehyde-3-phosphate dehydrogenase (GAPDH), 5′-CACCCTGTTGCTGTAGCCAAATTC-3′ (forward) and 5′-GACATCAAGAAGGTGGTGAAGCAG-3′ (reverse). PCR reactions were incubated at 94°C for 3 min, followed by 30 cycles of 94°C for 30 s, 57°C for 40 s, 70°C for 50 s, and then a final extension at 72°C for 10 min. The amplified products were separated by agarose gel electrophoresis on 2% gel and analyzed by BioImaging systems (UVP, Labworks, ver. 4.6).

### Lectin blot analysis

Cell lysates containing 30ug of proteins were subjected to 10 % sodiumdodecyl sulfate-polyacrylamide gel electrophoresis (SDS-PAGE). One gel was subjected to Coomassie Brilliant Blue (CBB) staining and another was transferred to nitrocellulose (NC) membrane (Pall Corporation). Then incubated with Biotin-labeled lectin Sambucus nigra agglutinin (SNA, Vector, 1:2000) and streptavidin-HRP (ZSGB-BIO, 1:4000) for 1h separately at room temperature. The detection was performed using enhanced chemiluminescence (ECL) kit (Advansta). Densitometry of proteins was analyzed with Gel-Pro software.

### Flow cytometry analysis for apoptosis

The apoptosis rate within each group of hepatocarcinoma cells were determined by using flow cytometry analysis with an Annexin V-FITC/PI apoptosis detection kit (Dojindo Laboratories). The cells were prepared with certain concentration of docetaxel (Sigma-Aldrich) for 24 h, harvested, washed twice with cold phosphate-buffered saline (PBS) and 1 × 10^5^ cells were resuspended in 100 ul 1× Annexin-V Binding Solution, and then incubated with Annexin V-FITC and PI solutions at room temperature for 15 min in the darkness before the addition of 400 ul 1× Annexin-V Binding Solution. The cells were analyzed by flow cytometry (BD Biosciences).

### DAPI staining

Formation of condensed chromatin and apoptosis bodies in hepatocarcinoma cells were quantified with 4′,6-diamidino-2-phenylindole (DAPI) nuclear staining. The cells were treated with certain concentration of docetaxel for 24 h and fixed with chilled 4% paraformaldehyde at room temperature for 20 min. The treated cells were then washed with cold PBS and stained with DAPI solution for 3 min in the dark. After washing with cold PBS, stained nuclei were imaged by fluorenscence microscope (Olympus Corp). The apoptotic ratio % = (apoptotic cells) / (total cells)× 100. Approximately 10 different fields of each sample were taken and analyzed within three independent experiments.

### Cell survival assays by cell counting kit-8

Hepatocarcinoma cells and its derivative cells, 2 × 10^3^ cells/well, were plated in 96-well plates. After an overnight incubation, cells were treated with 0, 0.01, 0.1, 1, 10 and 100μM of docetaxel for 24 h and 48 h. Cell counting kit-8 (CCK-8) solution (Dojindo Laboratories) was added to each well, and the plates were incubated at 37°C for 2 h. The absorbance at 450 nm was measured with a microplate reader (Thermo Fisher Scientific).

### Western blot analysis

Protein concentrations were measured using BCA kit (Beyotime). Equal amounts of proteins were subjected to 10-15% SDS-PAGE and transferred to NC membrane. Antibodies against ST6Gal-I (Abcam, 1:500), p38, pp38, caspase-3 or GAPDH (Bioworld, 1:300, 1:300, 1:400, 1:10,000), Bcl-2, Bax, Bad or PARP (Sangon, 1:500), ctochrome c or caspase-9 (Proteintech, 1:500) were used as the primary antibodies. The detection was performed using ECL kit. Densitometry of proteins was analyzed with Gel-Pro software.

### Statistical analysis

The quantitative data were presented as mean ± SD and experimental differences were analyzed by one-way ANOVA and two-tailed Student's t-test using SPSS 13.0 software. A value of *P* < 0.05 was considered as statistical significant. All experiments were performed in triplicate.
